# Dietary Polyamines Intake and Risk of Colorectal Cancer: A Case-Control Study

**DOI:** 10.3390/nu12113575

**Published:** 2020-11-22

**Authors:** Chu-Yi Huang, Yu-Jing Fang, Alinuer Abulimiti, Xia Yang, Lei Li, Kai-Yan Liu, Xin Zhang, Xiao-Li Feng, Yu-Ming Chen, Cai-Xia Zhang

**Affiliations:** 1 Department of Epidemiology, School of Public Health, Sun Yat-sen University, Guangzhou 510080, China; huangchy7@mail2.sysu.edu.cn (C.-Y.H.); abulimiti@mail2.sysu.edu.cn (A.A.); lilei65@mail2.sysu.edu.cn (L.L.); liuky9@mail2.sysu.edu.cn (K.-Y.L.); zhangx359@163.com (X.Z.); fengxli5@mail2.sysu.edu.cn (X.-L.F.); chenyum@mail.sysu.edu.cn (Y.-M.C.); 2 Department of Colorectal Surgery, Department of Experimental Research, Sun Yat-sen University Cancer Center, State Key Laboratory of Oncology in South China, Collaborative Innovation Center for Cancer Medicine, 651 Dongfeng Road East, Guangzhou 510060, China; fangyj@sysucc.org.cn (Y.-J.F.); yangxia@sysucc.org.cn (X.Y.)

**Keywords:** polyamine, putrescine, spermidine, spermine, colorectal cancer

## Abstract

Polyamines (including putrescine, spermidine, and spermine) are small, cationic molecules that are necessary for cell proliferation and differentiation. Few studies have examined the association of dietary polyamines intake with colorectal cancer risk. The aim of this study was to evaluate total polyamines, putrescine, spermidine, and spermine intake in relation to colorectal cancer risk in China. In total, 2502 colorectal cancer cases and 2538 age-(5-year interval) and sex-matched controls were recruited from July 2010 to April 2019. Odds ratios (ORs) and 95% confidence intervals (CI) were calculated by multivariable unconditional logistic regression after adjustment for various potential confounding factors. Higher intake of total polyamine, putrescine and spermidine was significantly associated with reduced risk of colorectal cancer. The adjusted ORs for the highest compared with the lowest quartile of intake were 0.60 (95% CI 0.50, 0.72; *P*_trend_ < 0.001) for total polyamines, 0.35 (95% CI 0.29, 0.43; *P*_trend_ < 0.001) for putrescine and 0.79 (95% CI 0.66, 0.95; *P*_trend_ = 0.001) for spermidine, respectively. However, higher intake of spermine was associated with increased risk of colorectal cancer, with an adjusted OR of 1.58 (95% CI 1.29, 1.93; *P*_trend_ < 0.001). This data indicate that higher intake of total polyamines, putrescine and spermidine, as well as lower intake of spermine, is associated with a decreased risk of colorectal cancer.

## 1. Introduction

Globally, colorectal cancer is the third most commonly diagnosed cancer and is the second leading cause of death from cancer [1]. It has been reported that obesity, genetic, lifestyle, and environmental factors were associated with colorectal cancer. Among these, diet plays a fundamental role in the incidence of colorectal cancer [2].

Polyamines are small, cationic molecules that are necessary for cell proliferation and differentiation. Dietary polyamines and their metabolites by intestinal microorganisms, as well as endogenous polyamines, have been shown to be major determinants of the total body polyamines pool [3]. Although cellular synthesis and microbial synthesis in the gut are also main sources for polyamines, biosynthesis of polyamines cannot meet the requirements to sustain cell growth [4], and the contribution of microflora in the gut to circulating levels of polyamines is still unknown at present. Human fecal polyamines concentration is reported to be associated with fecal microbiota [5], indicating that microbial synthesis of polyamines in the gut may be partly excreted in the feces. Animal experimental study showed that bacterially derived polyamines were not more important than those derived from diet [6]. Dietary polyamines are essential for the normal growth and development of the gastrointestinal tract. Putrescine, spermidine and spermine are three main polyamines found in prokaryotes and eukaryotes [7]. As with total polyamines, major sources of dietary putrescine and spermidine are fruits, vegetables and cheese, but spermine is rich in meat and grains [8,9]. Polyamines may serve as ligands at multiple sites on proteins, DNA, RNA, phospholipids, and nucleotide triphosphates [10]. The main biological functions of polyamines are regulation of gene expression by altering DNA structure and modulating signal transduction pathways [11,12]. Polyamines could regulate oncogene expression and function through transcriptional and posttranscriptional processes [13], playing an important part in inducing cell proliferation and differentiation [14]. Nevertheless, recent studies showed that high polyamine intakes might delay aging and inhibit 1,2-dimethylhydrazine-induced colon tumor genesis [15]. Moreover, putrescine mediated probiotics and affected longevity through directly inhibiting senescence in colonic cells [16,17].

So far, few epidemiological studies have reported the association between dietary polyamines intake and colorectal cancer risk. Only one prospective study examined the association between dietary polyamines intake and colorectal cancer risk in postmenopausal women [9]. An inverse association was observed between dietary polyamines intake and colorectal cancer risk in women, with some colorectal cancer risk-lowering behaviors in particular. However, this study did not include premenopausal women and men, and did not evaluate the associations between putrescine, spermidine and spermine and colorectal cancer risk. Thus, we conducted this study to estimate these associations among men and women residing in Guangdong province, China. Based on the experimental evidence of beneficial effects of polyamines on gastrointestinal health and longevity, as well as the protective results of epidemiological study, we hypothesized that higher intake of polyamines was associated with reduced risk of colorectal cancer.

## 2. Material and Methods

### 2.1. Study Subjects

This ongoing case-control study started in July 2010 and the design of the study has been previously reported [18]. In brief, cases were consecutively recruited from one teaching hospital affiliated to Sun Yat-sen University, Guangzhou, China. The inclusion criteria were as follows: 30–75 years of age, with a primary, histologically confirmed colorectal cancer diagnosed no more than 3 months prior to the interview, natives of Guangdong or having lived in Guangdong for more than 5 years. Participants were excluded if they could not understand or speak Mandarin/Cantonese or had been diagnosed with any other type of cancer other than colorectal cancer. From July 2010 to April 2019, a total of 2528 out of 2817 eligible cases were successfully interviewed, with a response rate of 89.74%. Twenty six subjects with extreme energy intake (<600 or >3500 kcal/d for women, and <800 or >4200 kcal/d for men) [19] were removed from the analysis. Finally, the present study included 2502 cases.

Cancer-free controls were frequency matched to cases by 5-year age group and sex. These came from two control groups. The first group of hospital-derived controls were recruited from the inpatients admitted to the Departments of Otorhinolaryngology, Vascular Surgery and Plastic and Reconstructive Surgery in the affiliated Hospital of Sun Yat-sen University during the same period as the cases. They were mainly admitted for medical conditions as follows: sudden deafness, sinusitis, otitis media, trigeminal neuralgia, facial paralysis, and varicose veins. Out of 1533 eligible hospital-derived controls, 1357 were successfully interviewed, a participation rate of 88.52%. The second control group was recruited through flyers, advertisements, invitations or referrals. A random selection occurred of 1181 community-derived controls by sampling from 3089 successfully interviewed community residents from a community-based cohort [20], matched to cases via 5-year age group and sex. In total, 2538 controls were included.

This study was conducted in accordance with the guidelines of the Declaration of Helsinki and was approved by the Ethical Committee of the School of Public Health, Sun Yat-sen University (No. 2019-018). All study subjects signed informed consent forms before the interview.

### 2.2. Data Collection

Information on demographic situation, lifestyle, body weight and height and family history of cancer was collected through face-to-face interviews by trained interviewers using a structured questionnaire. Detailed menstrual and reproductive histories were obtained from women. Clinical diagnoses and histological findings were abstracted from the hospital medical records. Body mass index (BMI) was calculated by dividing weight in kilograms by the square of height in meters. Regular smokers were defined as those who had smoked at least 1 cigarette per day for more than 6 months consecutively. Passive smokers were non-smokers who reported being exposed to others’ tobacco smoke for at least 15 min/day during the previous 5 years. Regular alcohol consumption was defined as alcohol drinking at least once per week during the past year. The intensity of physical activity was estimated based on occupational activity and household and leisure-time activities. The mean metabolic equivalent (MET)-hour value of each activity was calculated according to the Compendium of Physical Activities [21,22]. MET-hours/week was calculated as a product of the number of hours per week spent in an activity and the MET score of this specific type of activity. Postmenopausal status was defined as women who had cessation of menstrual periods for more than 12 months before interview.

### 2.3. Dietary Assessment

A validated 81-item food frequency questionnaire (FFQ) [23] was used to assess the habitual dietary intake of participants during the previous year, before diagnosis for case subjects or before the time of interview for controls. Each participant was asked for information on portion size and frequency of dietary intake which was used to calculate the intake of each food item in gram/day on average. Food photographs were provided to help participants to estimate the consumed amounts of food. Total energy and other nutrients intakes were calculated based on the 2002 Chinese Food Composition Table [24]. Special nutrient data, putrescine, spermidine and spermine, were calculated on the basis of published data from previous publications [25,26,27,28,29]. The total for polyamines was the sum of putrescine, spermidine and spermine.

### 2.4. Statistical Analysis

Comparison between cases and controls were performed using the *t*-test or Wilcoxon rank-sum test for continuous variables, and the χ^2^-test for categorical variables. The residual method was used to adjust for total energy intake [30]. Dietary total polyamines, putrescine, spermidine and spermine intakes were categorized into quartiles according to the distribution among controls for men and women separately. Odds ratios (ORs) and 95% confidence intervals (CIs) were calculated using multivariable unconditional logistic regression models, with the lowest quartile as the reference category. Linear trends for ORs were assessed. Based on comparison of basic characteristics between cases and controls, potential confounding variables included in the multivariable adjusted models were as follows: age, sex, residence, marital status, education, occupation, income level, smoking status, alcohol drinking, occupational physical activity, household and leisure-time activities, BMI, family history of cancer and energy intake. Fiber, calcium and vitamin D were related to colorectal cancer risk [31,32], and dietary fat and folate played a role in polyamine metabolism [33,34]. However, to avoid over adjustment, only total fat and Vitamin D were included in the multivariable regression models for the association between total polyamines, putrescine and spermidine intake and colorectal cancer risk, while dietary fiber, folate and calcium intakes were additionally adjusted for the associations between spermine and colorectal cancer risk. Age, BMI, household and leisure activities intakes of energy, total fat, Vitamin D, fiber, folate and calcium were included as continuous variables, whereas other confounding variables were regarded as categorical variables.

Analysis stratified by sex was conducted. The interaction was evaluated by including a product term in multivariable models. Subgroup analyses by cancer site (colon and rectal cancers) and different sources of controls (hospital-derived controls and community-derived controls) were also performed. All statistical analyses were done with SPSS 22.0 (SPSS, Inc., Chicago, IL, USA). All *p* values were two-sided and a *p* < 0.05 was considered to be statistically significant.

## 3. Results

Among the 2502 cases, 1425 were men and 1077 were women. In total, there were 1565 colon cancers and 937 rectal cancers. More cases than controls were married, lived in rural areas, and had active smoking, regular alcohol drinking and family history of cancer. Case subjects were also likely to have a larger proportion of farmers, less education, lower income, lower BMI, heavier occupational activity and fewer household and leisure activities. No significant differences were found in passive smoking between cases and control subjects. Age at menarche and menopausal status were also not significant differences between cases and controls among women (Table 1).

Compared with the controls, intake of energy-adjusted total polyamines, putrescine and spermidine was significantly lower among cases, and spermine intake was significantly higher among cases (Table 2).

As presented in Table 3, higher intake of total polyamines, putrescine and spermidine was significantly associated with reduced risk of colorectal cancer. The adjusted ORs for the highest quartile compared with the lowest quartile of intake were 0.60 (95% CI 0.50, 0.72; *P*_trend_ < 0.001) for total polyamine, 0.35 (95% CI 0.29, 0.43; *P*_trend_ < 0.001) for putrescine and 0.79 (95% CI 0.66, 0.95; *P*_trend_ = 0.001) for spermidine. However, higher intake of spermine was associated with increased risk of colorectal cancer, with an adjusted OR of 1.58 (95% CI 1.29, 1.93; *P*_trend_ < 0.001) comparing the highest quartile with the lowest quartile.

Analysis stratified by sex showed that intakes of total polyamines and putrescine were inversely associated with colorectal cancer both in men and women (Table 4). A significant interaction was observed only between the risk of colorectal cancer and putrescine intake, but not with total polyamines intake. In addition, the inverse association between spermidine intake and colorectal cancer risk and the positive association between spermine intake and colorectal cancer risk were observed only among men, with an adjusted OR of 71 (95% CI 0.55,0.91; *P*_trend_ = 0.002) for spermidine and 2.13 (95% CI 1.61,2.82; *P*_trend_ < 0.001) for spermine, comparing the highest quartile with the lowest quartile, respectively.

Subgroup analysis by cancer site showed that intakes of total polyamines, putrescine and spermidine were inversely associated, while spermine intake was positively associated with colon and rectal cancer risk (Table 5). Subgroup analysis by different sources of controls also showed no significant difference between dietary polyamines intake and risk of colorectal cancer when using either group (Table 6).

## 4. Discussion

This is the first study to evaluate the association between intake of total polyamines, putrescine, spermidine, and spermine and risk of colorectal cancer both in men and women. The results showed that higher intake of total polyamines, putrescine and spermidine was significantly associated with reduced risk of colorectal cancer. However, higher intake of spermine was associated with increased risk of colorectal cancer.

Dietary intake, cellular synthesis, and microbial synthesis in the gut are three main sources for polyamines in organisms. Cellular polyamines were polycationic compounds and could bind to negatively charged biomolecules, change their structures and modulate their functions [35,36]. Thus, cellular polyamines might affect signal transduction, translation, cell proliferation and differentiation [13,37,38,39]. Production of higher levels of polyamines by inducing intestinal bacterial groups may suppress low-grade colon inflammation and restore colonic barrier function [16]. However, another study showed that polyamines generated by bacterial biofilms in the colon may lead to inflammation and cell proliferation [40]. Dietary intake of polyamines is essential for the development and maintenance of the digestive tract [41]. Dietary polyamines can be absorbed and distributed to different tissues [42]. Most spermidine and spermine was rapidly absorbed without metabolism by intestinal cells, whereas a variable proportion of putrescine might transform into spermidine and other compounds [42,43]. Experimental study showed that approximately 61%–76% of three polyamines were absorbed after 10 min of administration in rat model, and there was no significant difference from each other [44].

Our observation of an inverse association between total polyamines intake and colorectal cancer risk was consistent with one prospective study which reported an inverse association of high polyamine intake with risk of colorectal cancer in postmenopausal women with BMI ≤ 25 [9]. Experimental animal models showed that polyamine suppressed low-grade colon inflammation, restored colonic barrier function, and protected against age-dependent memory impairment [16,17]. However, a randomized controlled trial reported that intake of total polyamines above the median was correlated with an increased risk of colorectal adenoma [45]. Similarly, higher polyamine intake was able to enhance the growth of aberrant crypt foci and increased the grade of adenoma dysplasia in mice initiated with a chemical colorectal carcinogen [46,47]. In addition, carcinogen-treated animals feeding with low polyamine diets had fewer aberrant crypt foci in the intestinal tract [4,46], but a high polyamine diet before carcinogen treatment decreased tumor incidence [15]. An animal study showed that increased polyamines intake was associated with a delay in chemically induced tumorigenesis [15]. Thus, dietary polyamines may be beneficial to inhibit colon carcinogenesis in low-risk colorectums yet promote tumor growth in colorectums once a cancer has developed. More studies, especially prospective cohort studies, are needed to examine the association between polyamines intake and colorectal cancer risk.

So far, no epidemiological study has reported the association between putrescine and spermidine intake and colorectal cancer risk. Our observation of inverse associations between putrescine and spermidine intake and colorectal cancer risk were supported by some animal studies. Autophagy can suppress oncogenesis through improving genomic stabilization, limiting inflammation, and facilitating adequate immune responses against cancer cells [48], and one animal study suggested that drinking water with spermidine supplement may enhance anticancer immuno-surveillance through autophagy [49]. Spermidine administration suppressed the growth of CT-26 colorectal tumors transplanted into immune-competent mice [50]. However, recent experimental study showed that putrescine contribute to Ca^2+^ channel remodeling in colorectal cancer and induce colon cancer cell proliferation and apoptosis resistance [51]. Some epidemiological studies showed that higher dietary spermidine intake was associated with lower incidence of cardiovascular disease [52] and all-cause mortality [53]. Due to the limited number of the study, more studies are needed to fully clarify this important issue.

The observation of a positive association between spermine intake and colorectal cancer risk in the present study is contrary to our initial hypothesis. Generally, ingested spermine could be quickly absorbed and distributed from the intestinal gut without degradation [54], and intake of spermine could influence blood spermine concentration in humans [55]. It was reported that high concentration of spermine might decrease the anti-tumor activity of DNA-binding anticancer drugs through its DNA-protecting activity in cancer cells [56]. Besides, increased blood spermine levels might play an important role in the suppression of normal anti-tumor cytotoxic activity, inducing cancer progression [57]. In addition, the originally discovered catabolic mechanism of polyamines was a two-step process, where spermidine and spermine were acetylated by the highly-inducible spermidine/spermine *N*^1^-acetyltransferase and were either excreted from the cell or oxidized by peroxisomal *N*^1^-acetylpolyamine oxidase (PAOX), resulting in H_2_O_2_ [58,59]_._ Spermine can also be oxidized directly by spermine oxidase (SMOX) to produce H_2_O_2_, which was localized in the cytoplasm and nucleus of mammalian cells [60]. Thus, the release of H_2_O_2_ produced by SMOX had greater potential for generating genetic damage than that produced by PAOX, which was produced in the presence of catalase in a normally functioning peroxisome [61]. Since spermine can exist within the cell at millimolar concentrations, the H_2_O_2_ produced by up-regulated SMOX is sufficient to evoke oxidative stress and DNA damage, which had been linked to subsequent carcinogenesis [61,62]. This specific catabolic mechanism might partly explain the positive association between colorectal cancer risk and intake of spermine, but not putrescine and spermidine.

Analysis stratified by sex showed that high intake of total polyamines and putrescine were associated with reduced risk of colorectal cancer in both men and women. A sex-modified interaction was found in the association of colorectal cancer risk with putrescine intake, but not with total polyamines intake. Besides, the inverse association of spermidine and positive association of spermine intake with the risk of colorectal cancer were observed only among men. One possible explanation for this sex difference may be the complexity of diet. A previous study suggested that differences existed in the dietary patterns of men and women [63], indicating that nutrients or food groups in men’s and women’s diets may be different and may have distinct effects on colorectal cancer. In the present study, the amount of spermidine and spermine intake in women was lower than that among men, which may in part explain the stronger correlation found in men. Furthermore, the sex hormone might be a potential factor that affects tumor carcinogenesis [64] and it was reported that estrogen and polyamine levels were correlated [65]. However, evidence is still limited and the mechanism of sex difference has not yet been fully clarified. Thus, modification by sex comparing spermidine and spermine intakes and colorectal cancer risk requires further investigation.

The present study has several strengths. This is the first to investigate the association between dietary polyamines intake and the risk of colorectal cancer both in men and women, and evaluate the association between putrescine, spermidine and spermine and colorectal cancer risk separately. A relatively large sample was included in our study, which provided adequate statistical power to detect associations with the risk of colorectal cancer. Moreover, a wider range of potential confounders were collected and adjusted in the multivariable logistic regression analysis.

Some limitations should also be considered. Firstly, selection bias is a common concern in case-control studies. In the present study, although colorectal cancer patients were recruited from only one hospital, patients at this hospital shared similar clinical characteristics with those from other large hospitals in mainland China [66]. Besides, the high participation rate of 89.74% for cases and 88.52% for hospital-derived controls in our study also helped to diminish selection bias. Secondly, recall bias was also of concern in case-control studies. To reduce this bias, we tried our best to interview cases as soon as the diagnosis was made. Photographs of usual food portion sizes were also used to assist subjects accurately quantify actual dietary intake. Thirdly, duplication of polyamines and other foods can interfere with the definitive distinction between individual effects. Fourthly, FFQ used in our study was only validated among women. However, the validation studies showed that there was no sex difference in the results of validity and reproducibility of FFQ [67,68,69]. Finally, the potential for dietary exposure misclassification and lack of more precise biomarkers of dietary polyamine remain concerns. However, such random measurement error tended to bias results towards the null, rather than towards spurious associations. 

## 5. Conclusions

The present study indicated that higher intake of total polyamines, putrescine and spermidine was associated with reduced risk of colorectal cancer, while higher intake of spermine was associated with increased risk of colorectal cancer in the Chinese population. More studies, especially prospective studies, are needed to further confirm the effects of polyamines intake on colorectal cancer risk.

## Figures and Tables

**Table 1:** Socio-demographic and clinical characteristics of colorectal cancer in the study population.

	Cases (*n* = 2502)	Controls (*n* = 2538)	*p*
Age (years); Mean ± SD	57.0 ± 10.3	56.7 ± 10.2	0.26
BMI (kg/m^2^); Mean ± SD	23.3 ± 3.2	23.6 ± 3.1	0.01
Age at menarche (years) ^a^; Mean ± SD	15.0 ± 2.4	15.0 ± 2.0	0.80
Household and leisure-time activities (MEET-h/week); Median (P_25_, P_75_)	28.9 (9.0, 52.5)	34.3 (16.3, 55.9)	<0.01
Sex; men; *n* (%)	1425 (57.0)	1425 (57.0)	0.94
Marital status; married; *n* (%)	2383 (95.2)	2383 (95.2)	<0.01
Residence; urban; *n* (%)	1612 (64.4)	1997 (78.7)	<0.01
Educational level; *n* (%)			<0.01
Primary school or below	789 (31.5)	567 (22.3)	
Secondary school	697 (27.9)	616 (24.3)	
High school	606 (24.2)	678 (26.7)	
College or above	410 (16.4)	677 (26.7)	
Occupation; *n* (%)			<0.01
Administrator/other white-collar worker	347 (13.9)	463 (18.2)	
Blue-collar worker	547 (21.9)	553 (21.8)	
Farmer/other	1608 (64.3)	1522 (60.0)	
Income (yuan/month, RMB); *n* (%)			<0.01
<2000	354 (14.1)	317 (12.5)	
2001–5000	836 (33.4)	935 (36.8)	
5001–8000	737 (29.5)	785 (30.9)	
>8001	575 (23.0)	501 (19.7)	
Smokers; *n* (%)	978 (39.1)	773 (30.5)	<0.01
Passive smoking; *n* (%)	682 (27.3)	750 (29.6)	0.07
Regular drinker; *n* (%)	450 (18.0)	359 (14.1)	<0.01
Family history of cancer; *n* (%)	362 (14.5)	145 (5.7)	<0.01
Occupational activity; *n* (%)			<0.01
Non-working	316 (12.6)	894 (35.2)	
Sedentary	697 (27.9)	533 (21.0)	
Light occupation	675 (27.0)	600 (23.6)	
Moderate occupation	390 (15.6)	238 (9.4)	
Heavy activity occupation	424 (16.9)	273 (10.8)	
Menopausal status ^a^; postmenopausal; *n* (%)	776 (72.1)	791 (72.2)	0.92

^a^ Among women subgroup.

**Table 2:** Intakes of polyamines and kinds of nutrients among colorectal cancer cases and controls.

	Cases		Controls		*p* ^a^
	Mean ± SD	P_50_ (P_25_, P_75_)	Mean ± SD	P_50_ (P_25_, P_75_)	
Energy (kJ/day)	1557.90 ± 481.89	1492.32 (1211.70, 1832.34)	1647.55 ± 526.75	1548.42 (1267.10, 1943.72)	<0.001
Fiber (g/day) ^b^	8.89 ± 2.86	8.57 (6.96, 10.40)	9.61 ± 2.71	9.30 (7.73, 11.17)	<0.001
Total fat (g/day) ^b^	34.23 ± 13.53	32.36 (24.37, 42.00)	34.73 ± 11.71	33.62 (26.81, 40.91)	0.002
Folate (μg/day) ^b^	216.00 ± 56.16	209.45 (178.06, 245.55)	229.40 ± 55.37	223.27 (191.77, 259.75)	<0.001
Vitamin D (μg/day) ^b^	5.67 ± 3.85	4.87 (3.16, 7.22)	6.54 ± 3.61	5.91 (4.07, 8.31)	<0.001
Calcium (mg/day) ^b^	404.14 ± 460.97	377.94 (292.80, 479.80)	451.79 ± 168.57	428.70 (332.37, 546.38)	<0.001
Polyamines (μmol/day) ^b^	170.20 ± 58.38	162.42 (130.31, 201.65)	188.89 ± 68.87	178.59 (142.18, 221.04)	<0.001
Putrescine (μmol/day) ^b^	56.19 ± 31.77	50.73 (36.77, 54.23)	73.08 ± 46.74	63.25 (45.78, 86.81)	<0.001
Spermidine(μmol/day) ^b^	68.44 ± 28.53	64.23 (48.60, 83.30)	72.23 ± 26.96	68.61 (53.52, 87.32)	<0.001
Spermine (μmol/day) ^b^	45.13 ± 13.20	43.81 (36.23, 52.81)	43.33 ± 12.41	42.51 (34.26, 51.14)	<0.001

^a^ Wilcoxon’s rank-sum test comparing median values among cases and controls. ^b^ The consumption was adjusted for total energy intake by residual method.

**Table 3:** Risks for colorectal cancer according to quartiles of polyamines intake (Odds ratios and 95% confidence intervals).

	Q1	Q2	Q3	Q4	*P_trend_*
Total polyamines					
Median intake (μmol/day)	114.51	157.26	193.13	254.52	
No. of cases/controls	895/634	652/635	536/634	419/635	
Crude OR (95% CI)	1	0.73 (0.63,0.84)	0.60 (0.51,0.70)	0.47 (0.40,0.55)	<0.001
Adjusted OR (95% CI) ^a^	1	0.70 (0.60,0.83)	0.61 (0.51,0.72)	0.55 (0.46,0.66)	<0.001
Adjusted OR (95% CI) ^b^	1	0.73 (0.62,0.86)	0.65 (0.55,0.77)	0.60 (0.50,0.72)	<0.001
Putrescine					
Median intake (μmol/day)	33.57	52.73	70.28	111.67	
No. of cases/controls	1031/635	679/635	543/634	249/634	
Crude OR (95% CI)	1	0.66 (0.57,0.76)	0.53 (0.45,0.61)	0.24 (0.20,0.29)	<0.001
Adjusted OR (95% CI) ^a^	1	0.71 (0.61,0.84)	0.59 (0.50,0.70)	0.33 (0.27,0.40)	<0.001
Adjusted OR (95% CI) ^b^	1	0.73 (0.62,0.86)	0.62 (0.52,0.74)	0.35 (0.29,0.43)	<0.001
Spermidine					
Median intake (μmol/day)	42.25	60.16	76.04	103.08	
No. of cases/controls	804/633	642/637	505/633	551/635	
Crude OR (95% CI)	1	0.79 (0.68,0.92)	0.63 (0.54,0.74)	0.68 (0.59,0.80)	<0.001
Adjusted OR (95% CI) ^a^	1	0.79 (0.67,0.93)	0.61 (0.51,0.73)	0.73 (0.61,0.87)	0.001
Adjusted OR (95% CI) ^b^	1	0.81 (0.69,0.96)	0.65 (0.54,0.77)	0.79 (0.66,0.95)	0.001
Spermine					
Median intake (μmol/day)	27.23	37.34	45.89	58.12	
No. of cases/controls	490/634	668/635	616/635	728/634	
Crude OR (95% CI)	1	1.36 (1.16,1.60)	1.26 (1.07,1.48)	1.49 (1.27,1.74)	<0.001
Adjusted OR (95% CI) ^a^	1	1.35 (1.13,1.61)	1.17 (0.97,1.40)	1.28 (1.07,1.53)	0.059
Adjusted OR (95% CI) ^b^	1	1.48 (1.23,1.78)	1.35 (1.11,1.63)	1.58 (1.29,1.93)	<0.001

^a^ OR was adjusted for age, sex, residence, marital status, education, occupation, income level, smoking status, alcohol drinking, occupational physical activity, household and leisure-time activities, BMI, family history of cancer and energy intake. ^b^ OR was adjusted for above confounders. Total polyamines, putrescine and spermidine additionally adjusted for total fat and Vitamin D intakes. Spermine additionally adjusted for dietary fiber, folate and calcium intakes.

**Table 4:** Associations between polyamines intake and colorectal cancer risk stratified by sex (Odds ratios and 95% confidence intervals).

	Men (*n* = 1425/1443)					Women (*n* = 1077/1095)					*P*_interaction_
	Q1	Q2	Q3	Q4	*P*_trend_	Q1	Q2	Q3	Q4	*P*_trend_	
Total polyamines											0.595
Median intake (μmol/day)	119.61	163.58	199.05	260.55		110.02	151.46	185.54	245.80		
No. of cases/controls	504/361	387/361	307/360	227/361		391/273	265/274	229/274	192/274		
Crude OR (95% CI)	1	0.77 (0.63,0.94)	0.61 (0.50,0.75)	0.45 (0.36,0.56)	<0.001	1	0.68 (0.54,0.85)	0.58 (0.46,0.74)	0.49 (0.39,0.62)	<0.001	
Adjusted OR (95% CI) ^a^	1	0.72 (0.58,0.91)	0.60 (0.47,0.76)	0.48 (0.38,0.62)	<0.001	1	0.68 (0.53,0.88)	0.62 (0.48,0.81)	0.63 (0.47,0.83)	0.001	
Adjusted OR (95% CI) ^b^	1	0.75 (0.59,0.94)	0.64 (0.50,0.81)	0.53 (0.40,0.68)	<0.001	1	0.72 (0.55,0.92)	0.67 (0.51,0.88)	0.68 (0.51,0.91)	0.006	
Putrescine											0.002
Median intake (μmol/day)	34.92	55.70	73.62	120.76		31.61	50.08	67.44	103.87		
No. of cases/controls	621/361	407/361	283/361	114/360		410/274	272/274	260/273	135/274		
Crude OR (95% CI)	1	0.66 (0.54,0.79)	0.46 (0.37,0.56)	0.18 (0.14,0.24)	<0.001	1	0.66 (0.53,0.83)	0.64 (0.51,0.80)	0.33 (0.26,0.43)	<0.001	
Adjusted OR (95% CI) ^a^	1	0.74 (0.59,0.91)	0.48 (0.38,0.60)	0.25 (0.19,0.33)	<0.001	1	0.66 (0.51,0.85)	0.73 (0.56,0.94)	0.45 (0.34,0.61)	<0.001	
Adjusted OR (95% CI) ^b^	1	0.76 (0.61,0.94)	0.50 (0.39,0.64)	0.27 (0.20,0.35)	<0.001	1	0.68 (0.53,0.88)	0.77 (0.59,0.99)	0.48 (0.36,0.66)	<0.001	
Spermidine											0.889
Median intake (μmol/day)	42.65	61.11	76.56	103.76		41.44	59.10	75.51	102.04		
No. of cases/controls	465/360	355/362	297/360	308/361		339/273	287/275	208/273	243/274		
Crude OR (95% CI)	1	0.75 (0.62,0.93)	0.64 (0.52,0.79)	0.66 (0.54,0.81)	<0.001	1	0.84 (0.57,1.06)	0.61 (0.48,0.78)	0.71 (0.57,0.90)	<0.001	
Adjusted OR (95% CI) ^a^	1	0.74 (0.59,0.94)	0.62 (0.49,0.78)	0.64 (0.50,0.82)	<0.001	1	0.84 (0.65,1.08)	0.62 (0.47,0.81)	0.84 (0.64,1.10)	0.051	
Adjusted OR (95% CI) ^b^	1	0.77 (0.61,0.97)	0.65 (0.51,0.83)	0.71 (0.55,0.91)	0.002	1	0.86 (0.66,1.11)	0.64 (0.49,0.84)	0.89 (0.67,1.18)	0.152	
Spermine											0.001
Median intake (μmol/day)	31.02	39.99	48.95	60.91		24.56	34.17	42.77	54.04		
No. of cases/controls	230/360	375/361	335/362	485/360		260/274	293/274	281/273	243/274		
Crude OR (95% CI)	1	1.63 (1.31,2.03)	1.45 (1.16,1.81)	2.11 (1.70,2.61)	<0.001	1	1.13 (0.89,1.43)	1.09 (0.86,1.38)	0.94 (0.73,1.19)	0.543	
Adjusted OR (95% CI) ^a^	1	1.60 (1.24,2.06)	1.23 (0.95,1.59)	1.64 (1.28,2.10)	0.004	1	1.21 (0.93,1.57)	1.07 (0.82,1.40)	0.89 (0.68,1.18)	0.293	
Adjusted OR (95% CI) ^b^	1	1.77 (1.37,2.30)	1.45 (1.11,1.90)	2.13 (1.61,2.82)	<0.001	1	1.33 (1.01,1.75)	1.25 (0.94,1.66)	1.10 (0.81,1.49)	0.761	

^a^ OR was adjusted for age, sex, residence, marital status, education, occupation, income level, smoking status, alcohol drinking, occupational physical activity, household and leisure-time activities, BMI, family history of cancer and total energy intake. ^b^ OR was adjusted for above confounders. Total polyamines, putrescine and spermidine additionally adjusted for total fat and Vitamin D intakes. Spermine additionally adjusted for dietary fiber, folate and calcium intakes.

**Table 5:** Associations between polyamines intake and risk of colon and rectal cancers (Odds ratios and 95% confidence intervals).

	Colon Cancer (*n* = 1565)					Rectal Cancer (*n* = 937)				
	Q1	Q2	Q3	Q4	*P*_trend_	Q1	Q2	Q3	Q4	*P*_trend_
Total polyamines										
Median intake (μmol/day)	114.76	157.27	192.82	256.22		115.36	158.34	196.18	259.10	
No. of cases/controls	527/634	421/635	354/634	263/635		368/634	231/635	182/634	156/635	
Crude OR (95% CI)	1	0.80 (0.67,0.94)	0.67 (0.56,0.80)	0.50 (0.41,0.59)	<0.001	1	0.63 (0.51,0.76)	0.50 (0.40,0.61)	0.42 (0.34,0.53)	<0.001
Adjusted OR (95% CI) ^a^	1	0.75 (0.63,0.91)	0.65 (0.54,0.80)	0.56 (0.45,0.69)	<0.001	1	0.63 (0.51,0.79)	0.56 (0.44,0.70)	0.56 (0.44,0.71)	<0.001
Adjusted OR (95% CI) ^b^	1	0.77 (0.64,0.93)	0.69 (0.57,0.84)	0.60 (0.48,0.74)	<0.001	1	0.66 (0.53,0.82)	0.61 (0.48,0.77)	0.64 (0.49,0.82)	<0.001
Putrescine										
Median intake (μmol/day)	34.03	52.65	70.28	112.24		33.63	53.47	71.59	115.10	
No. of cases/controls	617/635	446/635	343/634	159/634		414/635	233/635	200/634	90/634	
Crude OR (95% CI)	1	0.72 (0.61,0.85)	0.56 (0.47,0.66)	0.26 (0.21,0.32)	<0.001	1	0.56 (0.46,0.68)	0.48 (0.40,0.59)	0.22 (0.17,0.28)	<0.001
Adjusted OR (95% CI) ^a^	1	0.77 (0.63,0.91)	0.60 (0.49,0.72)	0.33 (0.26,0.42)	<0.001	1	0.65 (0.52,0.80)	0.60 (0.48,0.75)	0.35 (0.26,0.46)	<0.001
Adjusted OR (95% CI) ^b^	1	0.77 (0.64,0.93)	0.62 (0.51,0.75)	0.35 (0.28,0.44)	<0.001	1	0.68 (0.55,0.84)	0.64 (0.51,0.84)	0.38 (0.29,0.51)	<0.001
Spermidine										
Median intake (μmol/day)	42.41	60.18	76.03	103.40		42.70	60.55	77.05	104.01	
No. of cases/controls	476/633	415/637	320/633	354/635		328/633	227/637	185/633	197/635	
Crude OR (95% CI)	1	0.87 (0.73,1.03)	0.67 (0.56,0.80)	0.74 (0.62,0.88)	<0.001	1	0.69 (0.56,0.84)	0.56 (0.46,0.70)	0.60 (0.49,0.74)	<0.001
Adjusted OR (95% CI) ^a^	1	0.83 (0.69,1.01)	0.64 (0.52,0.78)	0.76 (0.62,0.92)	0.001	1	0.71 (0.57,0.89)	0.59 (0.47,0.75)	0.71 (0.56,0.89)	0.001
Adjusted OR (95% CI) ^b^	1	0.86 (0.71,1.04)	0.67 (0.55,0.83)	0.81 (0.64,0.99)	0.011	1	0.74 (0.59,0.92)	0.64 (0.50,0.81)	0.79 (0.59,0.98)	0.017
Spermine										
Median intake (μmol/day)	27.29	37.46	45.94	58.02		27.96	37.49	46.89	57.69	
No. of cases/controls	296/634	422/635	386/635	461/634		194/634	246/635	230/635	267/634	
Crude OR (95% CI)	1	1.42 (1.18,1.71)	1.30 (1.08,1.57)	1.56 (1.30,1.87)	<0.001	1	1.27 (1.02,1.57)	1.18 (0.95,1.48)	1.38 (1.11,1.71)	0.011
Adjusted OR (95% CI) ^a^	1	1.38 (1.12,1.69)	1.16 (0.94,1.43)	1.30 (1.06,1.60)	0.090	1	1.28 (1.01,1.62)	1.14 (0.90,1.46)	1.22 (0.96,1.56)	0.237
Adjusted OR (95% CI) ^b^	1	1.52 (1.24,1.88)	1.35 (1.08,1.68)	1.60 (1.28,2.01)	0.001	1	1.39 (1.09,1.78)	1.32 (1.02,1.70)	1.49 (1.14,1.94)	0.011

^a^ OR was adjusted for age, sex, residence, marital status, education, occupation, income level, smoking status, alcohol drinking, occupational physical activity, household and leisure-time activities, BMI, family history of cancer and total energy intake. ^b^ OR was adjusted for above confounders. Total polyamines, putrescine and spermidine additionally adjusted for total fat and Vitamin D intakes. Spermine additionally adjusted for dietary fiber, folate and calcium intakes.

**Table 6:** Associations between polyamines intakes and colorectal cancer risk by different sources of controls (Odds ratios and 95% confidence intervals).

	Hospital-Derived Controls (*n* = 1342/1357)					Community-Derived Controls (*n* = 1160/1181)				
	Q1	Q2	Q3	Q4	*P*_trend_	Q1	Q2	Q3	Q4	*P*_trend_
Total polyamines										
Median intake (μmol/day)	127.17	165.89	199.04	255.44		114.41	157.39	195.44	256.91	
No. of cases/controls	466/339	322/339	302/340	252/339		472/294	305/296	217/296	166/295	
Crude OR (95% CI)	1	0.69 (0.56,0.85)	0.67 (0.56,0.80)	0.50 (0.41,0.59)	<0.001	1	0.64 (0.52,0.80)	0.46 (0.36,0.57)	0.35 (0.28,0.45)	<0.001
Adjusted OR (95% CI) ^a^	1	0.74 (0.60,0.93)	0.73 (0.58,0.91)	0.67 (0.53,0.84)	0.001	1	0.58 (0.43,0.76)	0.45 (0.33,0.61)	0.36 (0.26,0.51)	<0.001
Adjusted OR (95% CI) ^b^	1	0.74 (0.60,0.93)	0.73 (0.58,0.91)	0.68 (0.53,0.86)	0.001	1	0.64 (0.48,0.86)	0.53 (0.39,0.73)	0.45 (0.32,0.64)	<0.001
Putrescine										
Median intake (μmol/day)	34.45	52.10	69.63	98.51		33.05	51.06	69.18	101.27	
No. of cases/controls	435/339	388/340	301/339	218/339		587/295	295/295	197/297	81/294	
Crude OR (95% CI)	1	0.89 (0.73,1.09)	0.69 (0.56,0.85)	0.50 (0.40,0.63)	<0.001	1	0.50 (0.41,0.62)	0.33 (0.27,0.42)	0.14 (0.10,0.18)	<0.001
Adjusted OR (95% CI) ^a^	1	0.96 (0.77,1.18)	0.82 (0.65,1.03)	0.63 (0.50,0.81)	<0.001	1	0.47 (0.35,0.62)	0.29 (0.22,0.40)	0.16 (0.11,0.24)	<0.001
Adjusted OR (95% CI) ^b^	1	0.99 (0.80.1.23)	0.85 (0.67,1.07)	0.67 (0.52,0.85)	0.001	1	0.49 (0.37,0.65)	0.31 (0.23,0.43)	0.18 (0.12,0.27)	<0.001
Spermidine										
Median intake (μmol/day)	45.37	63.06	80.42	106.32		43.94	62.79	78.77	103.31	
No. of cases/controls	436/339	349/339	284/340	273/339		372/295	270/295	248/296	270/295	
Crude OR (95% CI)	1	0.80 (0.65,0.98)	0.65 (0.53,0.80)	0.63 (0.51,0.78)	<0.001	1	0.73 (0.58,0.91)	0.66 (0.53,0.83)	0.73 (0.58,0.91)	<0.001
Adjusted OR (95% CI) ^a^	1	0.83 (0.67,1.04)	0.67 (0.54,0.84)	0.74 (0.59,0.93)	0.002	1	0.66 (0.49,0.88)	0.72 (0.53,0.94)	0.78 (0.56,0.96)	0.003
Adjusted OR (95% CI) ^b^	1	0.86 (0.69,1.08)	0.71 (0.56,0.89)	0.79 (0.62,0.97)	0.014	1	0.69 (0.41,0.83)	0.75 (0.51,0.85)	0.82 (0.59,0.99)	0.058
Spermine										
Median intake (μmol/day)	32.87	42.04	49.82	60.65		30.91	41.54	48.99	60.39	
No. of cases/controls	261/339	328/339	352/340	401/339		246/295	283/296	313/295	318/294	
Crude OR (95% CI)	1	1.26 (1.01,1.57)	1.35 (1.08,1.68)	1.54 (1.24,1.91)	<0.001	1	1.15 (0.91,1.45)	1.27 (1.01,1.60)	1.30 (1.03,1.64)	0.019
Adjusted OR (95% CI) ^a^	1	1.27 (1.01,1.60)	1.39 (1.10,1.75)	1.58 (1.26,1.99)	<0.001	1	1.13 (0.83,1.54)	1.08 (0.79,1.48)	0.99 (0.72,1.36)	0.542
Adjusted OR (95% CI) ^b^	1	1.26 (0.99,1.60)	1.38 (1.08,1.75)	1.59 (1.24,2.05)	<0.001	1	1.39 (0.99,1.92)	1.65 (1.18,2.31)	1.86 (1.30,2.66)	<0.001

^a^ OR was adjusted for age, sex, residence, marital status, education, occupation, income level, smoking status, alcohol drinking, occupational physical activity, household and leisure-time activities, BMI, family history of cancer and total energy intake. ^b^ OR was adjusted for above confounders. Total polyamines, putrescine and spermidine additionally adjusted for total fat and Vitamin D intakes. Spermine additionally adjusted for dietary fiber, folate and calcium intakes.
